# "The Hospital" Nursing Mirror

**Published:** 1899-09-02

**Authors:** 


### "The Hospital" Nursing Mirror.

Being the Nursing Section of "The Hospital."

[Contributions for this Section of "The Hospital" should be addressed to the Editor, The Hospital, 28 & 29, Southampton Street, Strand, London, W.C., and should have the word "Nursing" plainly written in left-hand top corner of the envelope.]

### Notes on News from the Nursing World.

### THE ROYAL RED CROSS.

Last Friday, at Osborne, four nurses were decorated by the Queen with the Royal Red Cross. Miss Leonora Maxwell-Muller, lady superintendent of the Indian Nursing Service, received the order in recognition of the interest she has always shown in her work, and for special devotion and competency in the discharge of her multifarious and responsible duties, and the particular pains she has taken in training British soldiers and Army Hospital Corps attendants in nursing duties; while Miss Sarah Clarke, Miss Mary Nutt, and Miss Minnie Powell, Army Nursing Sisters, became entitled to it by the devotion they have manifested to the sick in the field hospitals of the West African Frontier Force, and their courage in contending with the many difficulties which are the necessary accompaniment of European life on the Niger. Moreover, as the official record says, "their unfailing attention and cheerfulness has probably been the means of saving many lives, and has been the constant subject of praise from all ranks of white men, both Imperial, and of the Royal Niger Company."

### NURSE NUTT AND NURSING ON THE NIGER.

Nurse Nutt, who sailed on Saturday for the Niger from Liverpool in the s.s. "Jebba," with the view of resuming the work which she has proved herself so well qualified to perform, writes us in reference to a recent "Note." Miss Nutt points out that Miss Griffin, well known on the West Coast as Sister Dorothy, went to Lokoja, 500 miles up the Niger, seven or eight years ago, living at the mission house of the Church Missionary Society. She also refers to the fact that the three nursing sisters who have just been decorated with the Royal Red Cross arrived at Lokoja in March, 1898, and were there until the end of the year, when, as stated in an interview with Miss Nutt in these pages, they received orders to proceed to Jebba, 230 miles higher up the river. Miss Nutt mentions that on their arrival at Lokoja there was not a hut in the camp, that the Lokoja Hospital was built six months later on a ridge about fifteen minutes' walk from the camp and five minutes from the artillery, the native hospital standing behind the artillery camp. As to the natives in the Delta, Nurse Nutt describes them as cannibals, but observes that higher up the river they are more civilised. During the year she was at Lokoja and Jebba she does not remember any dead bodies being dug up and eaten, "although," she adds, "in the Delta, I believe, they still kill and eat each other, a white man being the more dainty morsel, when they can catch one."

### AN INTERESTING FUNCTION IN INDIA.

Miss M. E. Barker, of the Indian Nursing Service, has been decorated with the Royal Red Cross in India. As a rule, the presentation is made before the assembled troops in garrison, or by Her Majesty herself. On this occasion Miss Barker was decorated at Murree, the head-quarters of the Punjab command. Sir Power Palmer, commanding the forces in the Punjab, organised the arrangements, which were most effective. All officers resident at Murree assembled in full uniform to witness the ceremony. The scarlet of the staff and Dragoons contrasted with the drab of the Frontier force and the sombre black of the Rifle Brigade, combined with the brilliant costumes of the numerous ladies, made a vivid foreground to the dark pine trees which surround the General's residence. General Palmer, in a brief speech, explained the object of the ceremony, and signified Her Majesty's commands. In the course of his remarks, he said he was sure that they were all pleased to have met to do honour to Miss Barker, on whom Her Majesty had been pleased to confer the decoration of the Royal Red Cross for her services during the late campaigns on the frontiers of India. "The Red Cross," he continued, "is the highest distinction that any woman can obtain, and most of the ladies of our Royal Family are proud to wear it. It has also been conferred on Lady Roberts for the interest which she took in the nursing of our soldiers. In my own small experience I have had opportunities of seeing the valuable work that these good sisters performed in Egypt as well as in this country. I have seen how their devotion and care of the sick and wounded has enabled the medical officers to attend to duties which otherwise they could not have had time to efficiently perform; and I am sure that all here congratulate Miss Barker on the honour that has been conferred on her by Her Majesty." Lady Palmer then pinned on the Cross, which is the third of the decorations earned by Miss Barker, the others being the medal for the Tirah expedition, and a medal earned by saving the life of a lady patient from a burning hospital.

### PRESENTATIONS OF THE ROYAL RED CROSS AT MALTA.

An equally interesting ceremony has just taken place at Malta, the recipients of the Royal Red Cross in this case being Miss A. G. Mark, superintendent of nurses, and Miss G. M. Payne, nursing sister. In the course of his address upon the occasion, the Deputy Governor, who acted as the representative of the Queen, observed that the high distinction had been bestowed upon the nurses "in recognition of their arduous self-sacrifice, skilful nursing, and tender care for those officers and men who were wounded or fell sick in recent times in Crete." Subsequently, General Owen, having pinned on the crosses with a few words of congratulation to each Sister, turned again to the parade, and said: "On my own behalf and on yours, I wish these ladies, so highly honoured, all health and happiness for many years to come, and that they may, like ministering angels as they are, continue their noble work of tending the sick and wounded soldier."

### THE QUEEN'S NURSES IN IRELAND.

The presentation by the Countess Cadogan, at the Viceregal Lodge, Dublin, of badges and certificates to a number of members of Her Majesty's Institute for Nurses was a notable event. District nurses from every quarter of Ireland were present at the ceremony, and thoroughly enjoyed Lady Cadogan's garden tea, to which they were also invited. Miss Dunn, general superintendent of the Irish branch of the Institute, and her assistant, Miss Blackmore, marshalled the nurses, and so successful were all the arrangements that it is hoped the event will become an annual one. There are now 75 members belonging to the Institute in the sister island.

### THE NIGHTINGALE FUND.

The report for last year of this fund contains several items of interest. On January 1st, 1898, there were 44 probationer-nurses in the school at St. Thomas's Hospital, and 67 were admitted during the year. In the same period 25 were discharged as unsuitable, or left from other causes, and 35 completed their probationary year's training, 51 thus remaining in the home on December 31st, of whom 20 were paying probationers. Thirty-five probationer-nurses who completed their year of training were placed on the register of Nightingale nurses, and were taken on to the staff of St. Thomas's Hospital. It is stated that from the opening of the school in June, 1860, to the end of 1898, a total of 1,519 candidates have been admitted; and 899, after completing a year's probationary training, have been placed on the register of Nightingale nurses, and received appointments in St. Thomas's or some other public hospital or infirmary or district nursing institution for the benefit of the sick poor. The annual gratuity of £2, allowed by the regulations for the term of three years to the nurses who have completed a year's service satisfactorily in some approved hospital or other institution, was awarded to 94 nurses. As to the financial position of the fund the receipts were £2,157 10s. 3d., or a balance of £95 10s. 7d. over the expenditure. Miss Gordon, the matron, gives a satisfactory report of the class instruction which the probationers have received from the home sister, Miss Haig-Brown, and also speaks favourably of the training afforded to the probationers in the wards under the supervision of the sisters and staff nurses.

### NURSES WITH MONEY-BOXES AT RAILWAY STATIONS.

With reference to our note of last week, we gladly publish the following letter from the matron of Portsmouth Royal Hospital, and trust that the hope expressed by Miss Boulton will be fulfilled: "In reference to your remarks respecting the Portsmouth Hospital Saturday collection, I beg to say that none of the Royal Portsmouth Hospital nursing staff took any part in the collection. The disgraceful behaviour of the young women who masqueraded in such travesty of nurses' uniform was much commented upon, and there is reason to believe that such a method of collection will, in the future, be discontinued."

### CHELSEA HOSPITAL FOR WOMEN.

The nurses of this hospital are rejoicing in extra long holidays this year. The whole edifice is undergoing thorough repair and redecoration. The new operating theatre promises to be excellent when finished. There is plenty of light, and the walls are plastered with a patent cement called "Seagliola." This material has been employed in some of the wards at St. Thomas's, as well as in other hospitals. It has the appearance of marble after it is polished, and is said to be more durable. At any rate, the effect is very good. A large students' gallery has been built, receding back over the corridor, beneath which an anæsthetic chamber is in process of preparation. At the other side of the theatre a sterilising room is being fitted up. The work has necessitated the closing of the in-patient department for about ten weeks, but the out-patients have continued to attend until quite recently, and will begin to come again early this month. The electric light, new hot and cold water service, and a lift will all tend to make the nurses' lot easier. It is only a short time since the nurses acquired their new home, and little has been done to the quarters of those resident in the hospital beyond painting and cleaning their rooms. The matron is now back in town from her holiday, and ready to take up the reins as soon as the workmen quit her domain.

### THE LEAGUE OF ST. BARTHOLOMEW'S NURSES.

The honorary secretary of this league—Miss R. Cox Davies—sends us full particulars of the objects of the association, which was recently formed "for mutual help and union" among the past and present certificated nurses of St. Bartholomew's Hospital. The qualification for membership will be the certificate of the hospital, though the executive reserve power to elect a certain number of nurses, who, although not holding the certificate, have filled posts of responsibility in the hospital. The annual subscription is to be only half-a-crown, but it is proposed to form a benevolent fund, which will be maintained by subscriptions and donations; and the executive propose to grant sums, either as loans or gifts, to such members as may be in temporary difficulties. There will be an annual meeting for the transaction of business, which will be followed by a social gathering.

### AN ALLEGED ATTEMPT TO BRIBE NURSES AT THE SALISBURY INFIRMARY.

The Salisbury Board of Guardians have taken a very proper course in the case of a complaint made by three of the nurses of the infirmary. A member of an organisation called the "Christian Endeavour" desired, in contravention of the rules of the Board of Guardians, to read a portion of Scripture in the women's ward, and when the nurse referred him to the rules, and said she was responsible for their being carried out, he persisted. The nurse naturally told him that she should be obliged to report the matter to the master, whereupon he said, "Suppose I bribed you with a sovereign, should you then tell all that went on?" The nurse and her colleagues very properly resented this remark, and though the perpetrator afterwards declared he was only joking, and apologised for the "joke," the Guardians—before whom the question was subsequently raised—have prohibited him from visiting the workhouse in future. We know nothing about the "Christian Endeavour," but we should have thought it was the primary duty of any person associated with a religious organisation to conform to rules, and to refrain from insulting nurses, even as a joke.

### SHORT ITEMS.

In spite of the protest of a minority of two, the Worcester Board of Guardians have increased the salaries of the nurses from £25 to £30 each; and it is understood that an attempt will be made to provide them with better accommodation.—A successful effort has been made in Newport, Isle of Wight, to re-establish the district nursing society, and at a meeting last week the necessary annual sum of £90 was guaranteed.

### Gynæcological Nursing.

By G. A. Hawkins-Ambler, F.R.C.S., Surgeon to the Samaritan Free Hospital for Women; Assistant Surgeon to the Stanley Hospital, Liverpool.

(*Continued from page* 281.)

### Sponges and Dabs.

A surgeon who permits a nurse to prepare his sponges is either dangerously confiding in nature or has immense confidence in her capacity and conscientiousness. No part of her duties, if this be entrusted to her, is more urgent in importance, for no more fertile source of danger exists for the abdominal surgeon than the use of sponges that are not absolutely sterile as regards the presence of germs and filth. Sponges are of such fine texture, so intricate and full of traps for the hiding of dirt, that they are a constant source of uneasiness to us. Many surgeons have given up their use altogether for this reason. They use dabs, such as I shall describe presently. But no substitute for a sponge can equal the sponge itself for elasticity, absorbing qualities, softness, and ease of handling, and, if we can be certain it is aseptic, it were a pity to discard an agent it is impossible to adequately replace. I never use a sponge twice; I have new sponges for every operation, and those prepared by myself. Not that I lack confidence in my nurses, but I do not consider it a fair responsibility to throw on a nurse, who might be blamed for sepsis that could have been introduced into the case through half a dozen different channels. It will be well, however, to give one or two methods of preparing sponges for use, and I give Dr. Kelly's first, as it is about the best that can be adopted. The steps are as follows: (1) Lay the sponges in a stout cloth and pound sufficiently to break up grit and lime; (2) rinse with warm water ten or more times until it remains clear; (3) immerse in a muriatic acid solution (two drams to a pint) for twenty-four hours; (4) immerse in warm saturated solution of permanganate of potash; (5) decolourise by washing in a hot saturated solution of oxalic acid; (6) wash in lime water to take out the oxalic acid; (7) rinse thoroughly in plain sterilised water; (8) immerse in 1-1,000 solution of bichloride of mercury for twenty-four hours; (9) preserve until used in a 3 per cent. carbolic acid solution. The hands from step 4 onwards must have been thoroughly sterilised.

In my experience sponges sometimes rot after using the permanganate of potash solution. I usually find the following method sufficient for sponge cleaning: (1) Pound the sponges to break up grit and shake out sand. (2) soak them in a solution of muriatic acid in water, strong enough to taste distinctly acid; change the solution twice a day, and do this two or three days if there be time. (3) Wash repeatedly in hot sterilised water (the hands being sterilised the while) for a good three hours. (4) Soak in 1-30 solution of carbolic acid till wanted. If the sponges be new and the washing conscientious, the hands sterilised, and the water hot, this should be enough. But I scarcely think that anyone except the man who is going to do, and be responsible for, the operation can be trusted to do this duty with satisfaction; in others, I fancy, it would become somewhat perfunctory.

Whether sponges be used or not, and unless she have instructions to the contrary, the nurse will do well to prepare a number of dabs. These are made of gauze, eight layers in thickness, and of a size varying from eight inches by six to six inches by four. About eighteen of these should be prepared for an abdominal section. They should be boiled for an hour before use and kept in a solution of 1 in 20 carbolic acid. Smaller dabs made of Berlin wool rolled into balls and covered with gauze, or rolls of plain gauze, all sterilised and preserved as above, are very useful. They should not have any frayed edges, and must be well tacked together with thin silk or white thread. In an emergency the above can be boiled in the kettle with the instruments to be used at the operation. Two large gauze squares a yard long by half a yard wide and eight layers thick are often useful, and three or four soft towels must be also boiled ready for use at the operation, and kept in antiseptic solution till required. A number of smaller dabs are useful for handling utensils with after your hands have been prepared; but, though you will have carefully cleansed your hands a little while before an operation, you must give them another good scrubbing immediately before, and you will take pains to prevent contamination of dressings, &c., that have been sterilised.

### Operations in Private Houses.

You will sometimes be sent on in advance of the surgeon to prepare the patient for operation and select and arrange the room she is to occupy. This involves a good deal of responsibility, and while you must not distract a woman who is already trembling with apprehension by fussy interference you must not permit anything to come between you and the thoroughness of your work. You will look for a room that is of southern aspect, well ventilated, but not draughty, that is quiet, removed from the bustle of the household, but not so out of reach as to make service difficult and complicated. The room must be away from the immediate neighbourhood of water-closets and drain pipes, although you will carefully inspect these to see that no defect is present in the drainage. This should have been overhauled by the sanitary authorities beforehand. Sometimes this overhauling is found necessary just before an operation, and is undertaken while the patient is under treatment. This means setting free septic organisms, and an outburst of trouble for the patient. Where there is no time for delay in operations, it is not well to rake up defective drainage at the moment a patient is under your care; it usually does more harm than good. See that no collection of dirty linen, no ancient dustbin, no dusty lumber-room are near the sick chamber. This must have a good morning light (an attic window admirable), but you must have the room quiet, sunny, and as bright and cheerful as possible. Remove all furniture that is not absolutely necessary—curtains, carpets, mats, upholstery, brackets, pictures, &c.—anything not essential as furniture. If the room is not occupied for a day or two before use, so much the better. You must dust carefully with a damp cloth floors, tables, windows, window ledges, the tops of doors, and the walls. Scrub the floors thoroughly with carbolic soap. You may have to disinfect the room with sulphur fumes or formol, after which remove the paper from the crevices of the doors. In any case open the windows and expose all to the sunlight and air as long as you have time for. The floor should be scrubbed and dried the day before operation, and a fire kept in the room. Never use a dry duster, and leave no crevice unwiped. The bed should be narrow enough to enable the patient to be handled from either side, and broad enough for her to lie comfortably and, in the later days, obtain some change of position. A good hair mattress is best, and the ordinary hospital bed is excellent for this purpose. Two beds in the room are an advantage, so that the patient may be lifted in her sheet from one to the other for a change and to enable her bed to be made, and aired out of the room. The bed must be well aired. You will have a blanket, waterproof, and draw sheet under the patient, and the clothing must be all perfectly clean and aired. On the morning of operation the dusting must be repeated with a damp cloth and in great detail.

### Across the Seas.

### DIFFICULTIES WITH NATIVE PATIENTS AT LAGOS.—II.

THE day following my arrival at Lagos I was placed in charge of the native male wards, the matron looking after the Europeans and female native patients herself. We rarely had more than four or five Europeans in at the same time, and when they exceeded that number I generally gave a helping hand, especially if there was any night nursing to be done. We were on duty at seven a.m., and always tried to get through as much work as we could in the comparatively cool morning hours. The wards being unconnected, we had a great deal of walking to do in the open air. We breakfasted at eleven a.m. It was called breakfast, but was really a substantial luncheon, our little breakfast of tea, bread and butter, and fruit being before going on duty. From eleven to one was our own time, and I believe the whole of Lagos indulged in a siesta then for an hour or so. Even our Kroo boys would have been very indignant if they had been asked to do any work during that time. At half-past one the whole place was humming again, as the European patients dined at two p.m. Poor things! I was very sorry for them, especially the convalescents, for you cannot very well feed an invalid on tinned things, and there was very little else to give them except skinny chickens and doubtful eggs. I never ventured to give them a boiled egg, as very often I broke half a dozen before I could get a fresh one. Our cook, too, was not very proficient in the culinary art; in fact, I was so tired of seeing food badly cooked that when I came home for my six months' holiday one of the first things I did was to go to the National Training School of Cookery and take a course of practical plain cooking lessons. Nurses are more fortunate nowadays as they get cooking lessons as part of their training, and they must find them most useful. I soon got friendly with my black patients, particularly the little ones. A small Jebu about eight years old was a special favourite. He was not long in discovering that I had a *penchant* for him, and was quick to take advantage of it. Directly I appeared in the ward he would whisper "Oyinbo (white woman), come here," and seize my umbrella; this he would unfurl at once and sit proudly under it, feeling, I daresay, every inch a chief. But his delight was unbounded when I sometimes graciously lent him my red parasol. Who so proud as my Jebu then; he would insist on getting out of bed and stalk round the ward with the parasol wide open, scowling at the Hausa patients.

The Jebus and Hausas are rival tribes, and do not love each other. Each tribe (and we had members of several among our patients) has its own distinctive mark tattooed on their faces, that of the Jebus being particularly forbidding, three deep scratches about three inches long, perpendicularly, on each cheek, and the same horizontally on the forehead I think. Our resident medical officer used to take a wicked delight in putting a little Hausa boy—he was little more than a baby—into the Jebu's bed. Terrible was the row that followed, and I had to fly to rescue the Hausa baby from his enemy's clutches. The admiration of the Hausas—I very nearly wrote adoration—of their Inspector-General was unbounded. He was six feet four, and proportionately broad in the shoulders, and they regarded him with almost superstitious awe. *Apropos* of superstition, a funny episode occurs to me. We had a cupboard in the back verandah of D ward where I kept the native patients' clothes, such as they were, a string of beads and a yard or two of calico very often forming their entire costume; the Hausas of course wore uniform, as they are all "soldiers of the Queen."

One day, work being slack, I thought I would have a turn at tidying up the cupboard, which was getting into a chaotic state of confusion. I summoned the nurses to help me and we went on sorting them out until I came across a bundle, at the sight of which nurses and patients fled incontinently. In vain I called to them; they simply would not come back. At last I lost patience, and rather crossly ordered Macaulay, who was certainly the most intelligent nurse we had, to come back directly and explain. Macaulay approached in fear and trembling. The bundle of clothes belonged to a man who had died; he was a big medicine man, and there were a lot of "jujus" among the clothes, and it would be certain death to anybody who meddled with them. Such was Macaulay's explanation, and I could not induce him to open it, so I did it myself, the patients crowding round fearfully expecting me to drop down dead any moment. Such a queer collection it was—bones (finger bones I think), strings of beads, funny little brass rings and coins, and, biggest fetish of all, a green parrot's head. I threw them all away except the rings and coins; in fact, I wore one of the brass rings all day, after cleaning it up, just to show my patients how silly they were. They were very much surprised that I did not fade gradually away into an early grave. I think it was one of our own servants who a little later tried the effect of another juju on me. I had occasion to reprimand him for neglecting his work, and the next day I discovered a couple of brass rings and some other uncanny looking objects all tied together with a tuft of human hair reposing on my chest of drawers. It was too dirty to handle, so I took it up gingerly with a pair of dressing forceps and handed it to the cook. He looked quite scared, snatched it from me, and dropped it in the fire, saying it was one of the very worst jujus going. So I escaped a second time scot free. Our patients had the most ridiculous names. Sunday and Friday were very popular, but I think Bottled Beer ran them very close; then there was Best Man, Black Man's Trouble, Whisky, New Potatos, and Half Jack. A boy called Trouble wished to be taken on as steward by the chief medical officer, so he wrote him a letter, addressing him as "Dear Hopkins!"

There was quite a run of tetanus cases during my first six months; we could seldom find a scratch on them, and they were often put down to vegetable poisoning. I believe the natives frequently resorted to poison to get an enemy out of the way. Even the nurses occasionally would refuse to touch the food prepared by the hospital's cook if they had offended him previously, and they would work themselves into a state of abject terror over a common or garden stomach ache.

I have mentioned our Kroo boys more than once. They were a constant source of trouble and amusement to us. The matron and I were each allowed a boy, and we could never induce them to agree. "Missis, who be chief steward; other feller he no be smart boy, he be black bushman," the speaker himself being as black as Erebus. The greatest insult you can offer a Kroo boy is to call him a bushman. They come over in scores from the Kroo coast, working their passage out, and make very good domestics. Those who do not go in for domestic service work on board the numerous steamers, but they all return to their home in a year. In the case of servants, they accompany their masters who are returning to England as far as the Kroo coast, and after six months, board every ship returning to Lagos until they find them, and accompany them back to Lagos. They are terrible thieves, but in such a droll way. They rarely rob their master, but they do not hesitate to plunder anyone else for his benefit. Sometimes to my horror my boy would bring me certain of the matron's belongings, saying, "Missis, you keep him, I tief him for you." If I remonstrated, the reply was, "I dash him for you, missis"—dash meaning a gift, or, to give.

The matron and I were off duty every alternate day from four to nine p.m. If we wished to go out together we had to make a written application to the chief medical officer. It was never refused us, but the applications were never made unless the wards were light. I used generally to walk about a mile and a half to the beach. The sea breezes were most refreshing after a stifling day, and the exercise kept my liver in order. To get to the beach you had to cross a bridge over a creek. It was called Five Cowrie Bridge, as before it was built you were ferried over for five cowries. I christened it the Bridge of Sighs. It was only a few hundred yards from the hospital, but very few people had the energy to walk any farther. Instead, they sat there evening after evening bemoaning their wretched fate and sighing for the year of service to be over, actually worrying themselves into ill-health.

### A Nurse-Patient's Visit to Bad-Nauheim.

In compliance with medical advice, full of hope of cure, excited with the idea of a trip abroad, and filled with no little curiosity as to what the baths, Dr. Schott, and his treatment—about which I had heard so much—would be like, I started off for Bad-Nauheim. At twenty minutes past eight p.m. I left Charing Cross, arrived at Queenborough at ten p.m., and went on board the steamer. It was a lovely moonlight night, warm and still, the sea as calm as a pond. About four a.m. I regretfully exchanged the ship for the train. My invalid appearance and small amount of luggage saved me all Custom House bothers. About mid-day I arrived at Köln, utterly exhausted, hot and weary, very glad to rest at the Hotel du Nord. Next morning at ten a.m. I continued my journey to Frankfurt, where I had a rest and a good lunch, and left at half-past two p.m. for Bad-Nauheim, which I reached at four p.m., very glad the long, hot journey was ended, but feeling thankful for all the help and attention I had received everywhere from the officials.

I had engaged a room at the Hotel Bellevue, and found the genial, attentive host ready to receive me. The hotel is not a grand one, but it is very well situated opposite the park, and midway between the baths and the kursaal, and is very clean and comfortable.

Next day the great doctor came, gave a bathing-treatment card, with instructions written thereon, and many verbal orders, and sent one of his masseuses to give his resistance exercises twice daily. Unless very ill or very rich, the patients visit the doctor every third day, and not he them.

The routine of a patient's life at Bad-Nauheim is mostly as follows: Seven a.m., cocoa and rusks; 8 a.m., bath, every third day no bath, but visit doctor; 9 a.m., cup of milk and back to bed till 11, exercises followed by some light nourishment; 1, dinner. After a short rest patients lie, sit, drive, or walk in the open air. At 4 p.m., cocoa and rusks or milk, and in the open air till 6 p.m., if able; 7 p.m., supper; 8 p.m., exercises. Most rooms have verandahs and screens to enable the very weak to be in the open air.

When patients strictly carry out their doctor's orders in suitable cases the effect of the baths in heart diseases is simply marvellous. Some foolish people do themselves great harm by disobeying the doctor's commands, or by taking the baths without consulting a local doctor at all.

There are six bathhouses and three varieties of baths; the running effervescent are very powerful, and cannot be given without a doctor's order. The baths were first used in 1871; previously only the three mineral springs for drinking were known.

Patients should never go to Bad-Nauheim by themselves if suffering from a heart affection. There were two when I was there, very bad and all alone. Everyone was very sorry for them, and very good to them.

I was favoured with fine weather most of the time, but found the place rather stuffy, probably owing to the exhalations from the hot springs. I enjoyed sitting out in the park, or on the terrace, listening to the band and watching the people, or driving up the wood-clad Johannisberg, where an extensive view of the surrounding country may be enjoyed. Another drive was to the Salinen. These are great erections of brushwood, to the top of which the saline waters are pumped. Salt collects by evaporation, and is then prepared for export. Around the erections are wooden verandahs, with seats where people sit to inhale the saline evaporations

Watching the Hessian peasants in their quaint garb in the park on Sundays interested me greatly. It was very nice to find a bath-house, hospitals, and good arrangements made for the treatment of very poor patients, adults and children.

An English chapel is to be built when sufficient money is collected, but at present services are held in a German church. Speaking and understanding German and French, I got much amusement and information out of my neighbours at table d'hôte in the Kursaal.

After five enjoyable weeks I returned home with a new lease of life, feeling very grateful to Providence for these wonderful waters, and to the clever physicians who know how to use them for the benefit of suffering humanity.

I may add that the Bad-Nauheim baths and Dr. Schott's exercises can now be given wherever there is a doctor who has been to Nauheim, and studied the treatment, and a nurse or masseuse who has learnt how to carry it out. The ingredients, chemically prepared, are sold in London. The baths can only be given in a porcelain or painted wooden bath large enough for immersion up to the neck. There are many doctors and nurses in Great Britain who understand and have learnt the treatment. This is a great advantage to those who are unable to go abroad.

### Minor Appointments.

Wrexham Joint Fever Hospital.—Miss H. G. McKendrick has been appointed Staff Nurse. She was trained at Delancey Fever Hospital, Cheltenham, and has since been assistant nurse at Corporation Hospital, Bootle; assistant nurse, City Hospital South, Liverpool; nurse at Stroud Road Hospital, Gloucester, during the recent epidemic of small-pox; nurse at Cardiff Sanatorium; assistant nurse at Eston Sanatorium; and nurse at the Isolation Hospital, Mogden, Isleworth.

Suffolk General Hospital, Bury St. Edmunds.—Miss Grace Horsman has been appointed Staff Nurse of the male wards. Miss Horsman has just completed three years' training at the Clayton Hospital, Wakefield. She had previously two years at the Hospital for Paralysis and Epilepsy, Queen's Square, London, and holds a certificate for massage and electricity.

Guest Hospital, Dudley.—Miss Barbara Powne has been appointed Sister in charge of the women and children's wards. She was trained at Guy's Hospital, and has since been district nurse at Manchester, sister at Northampton Hospital, and sister of the hospital and convalescent home at Swanley.

Suffolk General Hospital.—Miss Lilian Heather has been appointed Staff Nurse of the female wards. She has just completed her training at the Suffolk General Hospital.

### A Paris Hospital.

### HOW FRENCH PATIENTS ARE FED.

By an Occasional Correspondent.

The subject of feeding patients is necessarily interesting to the matrons and sisters of English hospitals. When I was in Paris recently I took the opportunity of obtaining some information about this branch of hospital management. I remember so well my student days and the peremptory ways of the sister the moment the dinner hour had come, and the hurry in which my clinical notes had to be ended and I to depart, that it was not without some little hesitation that I followed an old man and a strapping lad carrying large metal pails which betokened dinner at the Hospital of La Charité.

It was a lovely morning, but blazing hot, and I had already spent an hour in a surgical ward watching the dressings. I was now sitting in the garden, under a balcony, watching the sparrows enjoying a cool bath in the fountain which was playing in the centre of the square. Several white nightcapped men in blue-black cloaks were up on a top balcony looking down upon me and upon the fountain, and the old square was as quiet and beautiful as a little garden of peace.

It was only eleven o'clock, and the sun beat down upon the wide stone steps which led up from the one side of the square to the first balcony. Inside, the stairs were oak with iron balustrade and hexagonal tiles at the landings, but the oak was worn and the iron was dingy, and the tiles wanted relaying. The staircases were wide, and reminded me of the fine old oak staircases at Bart's., but they were badly kept, and the walls wanted painting, and the comparison was in favour of Bart's.! Following my guides up the stairs to the first landing I kept close to them as they passed into the wards through the swing doors.

There was no sister sitting at a table ready to glare upon me as I went in. There was no self reliant nurse or diffident pro. ready to come up and ask me what I wanted; no one took any notice of me as I crossed the red-tiled ward and across another little ward into the ward kitchen. There was a motherly-looking body dressed in rusty black with a homely cap and black ribbons in it, who was in the second ward as I passed through, and to whom I bowed and said "Good morning."

I had not been in the ward kitchen many minutes talking to the lad as he undid his cans and put the various things on the stove to keep warm, before this woman came in. Her face was very red and round and motherly, and she was very stout, and I fell to wondering why cooks were always so fat, for I had put her down as a sort of cook charwoman. She peeped into one pan and said "omelette," into another and cried "le foie," another was "cutlets," another "soup," and a last one "tapioca." She now lifted the omelettes out on the table and began cutting them into slices while the lad arranged the plates on a wagon, with the various dishes all ready for the different foods. When she had counted her slices of omelette twice over to make sure she had cut them up into the right number for her patients, and had told me confidentially that the biggest slices were for some soldiers whom she had in the ward, everything was put on the wagon, and the boy wheeled it out into the first ward. He then took the soup to those very sick patients who were on what was called the "first degree" dietary, and we went on pushing the wagon.

My stout friend now began to call out—in French, of course—"those who want their dinners come this way," and in a quiet, respectful little group the men soon surrounded us. Addressing them by their numbers, she gave to each on one plate a piece of the meat—*le foie*, that is, liver—and on another plate a slice of omelette. To those who were on the first degree dietary, and were yet not so bad as to be on soup alone, she gave out a cutlet and a little tapioca. It was a medical ward, and nearly all of the patients were up, and came to get their dinners in person; but in a few cases the patient asked for his own dinner and also for that of a comrade, who was presumably too ill to come up for it. Into each of these three joined wards the wagon was wheeled and the same thing gone through.

I discovered somewhat later, with a considerable shock, that the cook-charwoman was no less a person than *la surveillante*, a post which corresponds pretty nearly to our "ward sister."

Fancy taking one of the sisters in a large London hospita for the cook or the scrubber!

When all were served, I went and sat down by a young fellow suffering from phthisis, and, with him and his comrades on either side, discussed hospital dietary in general.

"Just tell me," I asked, "when you eat and what you eat?"

"Our first meal," he replied, "is at seven a.m."

"Yes, and what do you have?"

"Soup," he replied.

"Yes, and a piece of bread," put in his neighbour.

"Oh! yes, a small slice of bread, but nothing else."

"Soup and bread at seven a.m., then, that's right so far, is it?"

"Yes, Monsieur, quite right."

"Well, when do you eat next?" I continued.

"Oh, this meal, at half-past eleven, which consists, usually, of a slice of meat, some vegetables, and a piece of bread."

"Don't forget the wine," again chimed in our neighbour.

"Who ever does forget the wine? Monsieur sees for himself that we have wine," he retorted sharply; "but he sees that we have only a little, and could drink more if he were good enough to send some."

At this juncture the sister came round bringing another cup of wine apiece in honour of the *Fête Nationale*, so that my friend smoothed down his ruffled temper, took a drink, and went on to tell me that they had fish once or twice a week, and meat on every other day beside, that they had practically no fresh vegetables, but that dried peas and beans and lentils and haricots and potatoes were the stock routine, with omelette—as to-day—replacing the vegetables once a week or so.

"Being a Roman Catholic country," I asked, innocently, "I suppose Friday is always one of your fish days?"

The little peppery neighbour waxed hot at once. "Fish on Friday; non, monsieur, why should we? It is only a superstition, this fish on Friday! Le bon Dieu doesn't make our stomachs different on Fridays. Le bon Dieu lets us be ill on Fridays, so why shouldn't we eat something to make us strong on Fridays? Fish on Fridays; pah, talala, we don't believe in superstitions in Paris now!"

I was not anxious to start a theological discussion, so I went on, "Don't you have any pudding?"

"Non, monsieur."

"Nor pies?'

"Non, monsieur."

"Nor fruit?"

"Ah, no."

"But I see a man over there eating an apple."

"Oh, yes; his friends have brought it. Friends may bring things, but otherwise we never have fruit, either fresh or stewed."

I marvelled at a dinner so highly nitrogenous and stimulating—liver, omelette or legumens, wine—and went on to ask about the next meal.

"Our next meal is at five, and it is the same as this."

"Ah, but," I asked, "what do you have at tea-time?"

I could not at once make them quite understand what I meant, and it was still longer before I grasped that tea-time was an English custom, and not a French one, and that in La Charité hospital a patient had a soup breakfast and two meat dinners in the day, and that is all!

No tea, no coffee, no chocolate, no fresh vegetables, no puddings, pies, or fruit, no bread and butter, no toast! I began to think that I shouldn't like to be ill in Paris.

"But what do you drink?" I enquired.

"Wine, monsieur."

"And coco," put in my talkative neighbour.

"Oh," I replied, "you get cocoa. Well, that sounds nice and homely."

"Ah! non, monsieur, not cocoa, but *coco—'a reglise*."

I could not make it out; my French did not run to these words, until one man gave me his jug to taste, and I tasted—liquorice water!

Liquorice water is provided *ad lib*, and a capital thirst-quenching drink I should imagine it to be.

To ensure the accuracy of my facts I later on visited the kitchen and made a copy of the menu for the day as hung up for the cook's direction. It was as follows:—

MENU DU VENDREDI, 14th Juillet, 1899.

Malades.

Matin,* 1st degree—Potage, tapioca, rotis, cotellettes. 2nd degree—Rotis, riz au lait. 3rd and 4th degree—Foie de veau, sauce piquante, œufs en omelette.

Soir,† 1st degree—Potage, tapioca, œufs au lait. 2nd degree—Bouilli, haricots vert. 3rd degree—Rotis et salade.

‡ Personnel.

Matin, 1st degree—Cotellettes de veau, Lapins sauté et sauce Chasseur, salade, melon, riz au lait, potage. 2nd degree—Lapins sauté et sauce Chasseur, salade, gateau, melon.

Soir, 1st degree—Cotelletes de veau, riz au lait, legumes. 2nd degree—Rosbif, legumes, soup à l'oignon.

* That would be Eleven to Half-past Eleven a.m.
† That would be Five p.m.
‡ That is "the staff," which consists of two grades or degrees, which we may call senior and junior or upper and lower staff.

### The Nurses' Bookshelf.

Some Thoughts About Nursing. A Lecture to Nurses of the Metropolitan Hospital. By Oswald Browne, M.A., M.D., F.R.C.P. (George Allen, Ruskin House. Price 6d.)

There is much that Dr. Oswald Browne has to say in the course of his lecture that may well make the reader reflect. It is eminently practical, at the same time holding up a high ideal of work without which our best efforts must ever fall short of success. Observation rightly occupies a considerable part, for the habit of accurate and ready observation is one of the most important attributes of a nurse. Mutual forbearance, patience, and tact are also important qualities, and for these, as he tells us, there is a continual demand. Dwelling on what constitutes a really good nurse, we find on page 9 the following definition given by a great physician, "Implicit obedience. The responsibility is always ours." We should recommend every nurse to read, mark, learn, and inwardly digest the practical truths so unpretentiously set forth in this little work, for she will attain a higher standard boys doing

A Literal Fact.—In St. Helens the other day two women of the working class were overheard discussing a medical point. The husband of each had been very ill with pneumonia. Said one: "The doctor says as how it's th' worst case of pew-monia what ever recovered." "Eh! how strange," replied the other; "that's what they said about my mon, only they called it new-monia." "Ah! well, they're th' same thing," said the first speaker. "Oh! nay, they're not," was the reply; "in one o' 'em they spits blood." "Well, now," exclaims the other, "I wonder at 'em givin' 'em names so much alike. It bothers us poor olk."

### Nursing Organisation in New South Wales.

For some time past a movement has been on foot in Sydney, New South Wales, for the better organisation of trained nursing, and meetings of doctors, matrons, and nurses have been held with the object of promoting some scheme of constitution which should protect the profession of nursing from the competition of untrained women. The advisability of establishing a branch of the Royal British Nurses' Association has been discussed, but eventually it has been decided to form a separate association, to be known as the New South Wales Trained Nurses' Association.

An important meeting, largely attended, was held on July 21st, presided over by Dr. Graham, who explained the scheme of constitution drawn up by the committee appointed to consider the matter. The four cardinal objects of the proposed association were to promote the interests of trained nurses in all matters affecting their work as a whole; to establish a system of registration for trained nurses; to afford opportunities for discussing subjects bearing on the work of nursing; and, lastly, to initiate and control schemes that will afford to nurses a means of providing an allowance during incapacity for work caused by sickness, accident, age or other necessitous circumstances. In regard to qualification the committee had thought it wisest to follow the practice of the British Association for Nurses, namely, that candidates for registration must produce proof that they had been engaged for three years in work in hospitals or infirmaries recognised by the council, of which not less than 12 months must have been spent in a general hospital, containing at least 10 beds. In addition, references as to conduct and professional competence were required. Concessions, however, were proposed in the case of a nurse trained at a public hospital and holding her certificate, also in the case of a nurse trained in a private hospital containing not less than 10 beds, for three years, and holding a certificate from two reputable medical men. In such cases nurses would be eligible for membership up to January 1st, 1900, subject to the approval of the Council. In conclusion, he thought the proposed association was a principle of union which would be of material benefit to the nurses in the colony.

The question of qualification for registration, particularly in a retrospective sense, caused some warm debating. The ordinary qualification was eventually fixed at three years in a public hospital of not less than 40 beds, the retrospective limit being settled on a division (60 to 46 votes) as follows: That up to January 1st next nurses should be eligible for membership who had been two years in a training school or five years in a private hospital under a matron holding a certificate from a training school. Most of the medical men present held that this was hardly a sufficient concession, and that many competent nurses would be injured professionally in consequence, a feeling which has been gaining ground since the meeting, and is likely to find further expression, with the result that some modification will probably eventually be proposed.

The officers of the new association were duly elected at the meeting. Dr. Manning becomes the president, and Dr. Goode the vice-president. The hon. secretaries are Dr. Mills and Miss McGahey, matron of the Prince Alfred Hospital; and the hon. treasurer, Sir James Fairfax. A council of eighteen members was elected, consisting of two honorary members, four medical men, six matrons, and six nurses.

### Wants and Workers.

The Matron of the Basford Sanatorium, Notts, will be grateful for any contributions, such as books or toys, for the patients. All parcels will be acknowledged.

"F. S." writes: "I am not a nurse myself, but I take The Hospital, and shall be very pleased to pass it on to 'A Poor Nurse' at Parochial Hospital, Carrickmacross, co. Monaghan, when I have read it, and will write to her if she will send her address to F. S., Post Office, Bury St. Edmunds, Suffolk." Sister Leavis, Private Hospital, West Hartlepool, also kindly offers to post her copy of The Hospital every Tuesday evening.

### Echoes from the Outside World.

### AN OPEN LETTER TO A HOSPITAL NURSE.

As I write, the wind is blowing a big gale, the moon has departed, leaving a thick darkness on every side, and the rain is pouring steadily down. It is difficult, after this long drought, to believe that at last the much-desired change has come, and on Sunday, when the first drops of moisture fell, it was quite comical. Some one found a round damp spot on their cuff, followed by the discovery that a similar spot had fallen on someone else's glove, then we all looked at each other simultaneously, and exclaimed, in our surprise, with bated breath, "Why, it must be rain." As we hurried along, lest the storm should overtake us, I almost unconsciously began to hum fragments from the oratorio of "Elijah," for the scene brought to my mind so forcibly the stirring music of Mendelssohn. It is not easy to grasp the idea of a water famine, except one lives in the East End of London; but even unobservant souls staying in the country and looking at the cracking earth, the dusty roads, and the thirsty root crops, must realise, to some extent, the blessings of the temporary break-up of the fine weather.

Amongst our other expeditions this week we went over to Hayling Island. I had often been told of this particular seaside resort by friends, and their opinions had varied so much that I felt curious to ascertain for myself whether it was the "delightfully rural, quiet spot" which I had heard it called by those who liked it, or the "God-forsaken place," "the very last bit of earth created," the description accorded to it by its detractors. We found it took us almost as long to get to Hayling Island from Southsea as if we had gone to London, because there are two changes, and at both junctions the great fun on the part of the officials seems to be to let a train come in on one platform, allow the passengers time to jump out of their carriages and begin to ascend the bridge, and then, just as they appear in sight, give the signal, and send the connecting train off under their very noses. There is generally plenty of time before the next is due to allow the tempers of irate passengers to cool down. Between Havant and Hayling for some little distance the train seems to have turned suddenly into a steamer, for the bridge which connects the island with the mainland is only wide enough to contain a single line; therefore, when the tide is high, the water comes up quite close to the carriages, and gives the idea of the passenger being entirely surrounded by the sea. When the tide is out, then alas ! it is only a broad expanse of mud with here and there a glimpse of a heron fishing for his dinner to break the monotony of the scene.

Arrived at Hayling, we made up our minds to go through the village to the sea, as we had been told there were two roads we might take. After a few minutes we came to an hotel, a big barn and straw yard, and a small grocer's—under whose roof the post office had also taken refuge—with a butcher's next door. Some of our party suggested that we had come the wrong way. Accordingly, seeing a juvenile errand boy, I boldly accosted him and asked if we were right for the village. "This is all the village ye're likely to see," was the rather contemptuous rejoinder, and he passed on, wondering apparently what we found to laugh at. However, the youth had somewhat maligned the place, for after wandering along a lane which boasted a boarding-house and a hall for entertainments, we came towards the sea front, and there found half-a-dozen more shops. And wonderful shops they seemed to my London mind, for they appeared to stock everything possible in an incredibly small space, and to keep really quite a decent collection of necessities, and odds and ends. Between the shops and the sea is a common, interspersed with patches of shingle, which must be traversed to get down to the water. Most folks rush over on their cycles, and are lucky if they do not occasionally "spill" into a gorse or a bramble bush. There is a bath-house, where a hot sea bath may be had for 1s. 6d., if ordered beforehand, also a cup of hot tea, but from what I hear it is a little too much to expect both prepared on the same day. I conclude, the days that visitors have a bath at Hayling they partake of tea in the privacy of their own apartments. Those who bathe in the sea do so from machines one end of the island, and from tents erected on the common at the other. The bathing, I am told, is splendid, and if I remained in Hayling Island I should creep away from the end where the shops, the hotels, and the fashionable folk (!) do congregate, and stay at the other end, if possible, where the sea and the common seem all around, and everything is as unconventional as possible. The lanes are sweet, the wild flowers beautiful, and the peace and rest must be delightful for those who simply want nothing to do—and no one to see them do it.

But though we are so far away in the wilds of the country, to my surprise and sorrow I came across a nurse. You will wonder why such a sight should cause me surprise and sorrow. Because, my dear, the specimen I saw patrolling one of these country lanes was the most untidy of her species upon whom I have ever set eyes. She carried a young baby, who was, I should fancy, by the strange way it was laid out straight upon her arms, not by any means a strong little person. She talked to it lovingly and smilingly, and showed that a kind heart lay under the sadly untidy exterior. She wore a white dress which trailed along the road, causing a cloud of dust to rise around her at every footfall, smothering both herself and her charge. The constant friction had also caused the hem of the garment to give way, and here and there it hung in ragged festoons. Her semi-white collar and cuffs matched the dress well, showing distinct signs of wear. Her abundant fair hair threatened descent at each step, and the long grey veil which hung from her nurse's bonnet had caught in the hedge now and then, and had not emerged from the conflict without sundry tears and rents. I could not help wondering if she were only a "sham," donning the guise of a hospital nurse so as to secure, perhaps, higher wages, or whether she had indeed undergone three years' training and been the constant despair of some poor Sister throughout the whole of the time.

The oyster season opens with the first of September, and those who are fond of the succulent bivalve will rejoice to hear that the outlook is the best which has been seen for forty years. Apparently, no weather is too hot for oysters; they love to get as warm as possible and keep warm, and it is the constant changes of temperature with which, as a rule, we are favoured that is so fatal to their well-being. Whether the fact that they will be plump and delicious this winter—should prophecy be correct—will make any distinct impression upon the number consumed yet remains to be seen. Oyster growers are flattering themselves that this year at least they will be able to make their industry pay, but I am afraid that the prejudice against the bivalve in many minds is too strong to be easily uprooted. In so many instances typhoid fever seems to have been caused by eating oysters from polluted beds that it is no wonder timid people are fearful. On the South Coast I was assured the other day by a visitor that I could get lovely oysters for 3s. 6d. à 100, and I should be silly if I did not arrange to secure some later on when I returned to London. Almost the next day a native of the same place warned me not to touch the oysters from the beds hard by, as they were not safe. Those who crave much for the luxury for themselves or for invalids who can fancy nothing else can, however, generally find out which beds are supposed to be quite free from infection, and by sending direct avoid the risk of being supplied with oysters which have perhaps been purchased cheaply by the vendors, because they are of doubtful character.

### Typical Patients.

### THE NERVOUS PATIENT.

Mr. Standish was a long, lean man, with a mouth that was always in movement, and thick eyebrows which arched and straightened themselves incessantly. Of course, his first thought was to inform me, with much care and elaboration, that he was not a nervous man, but his actions contradicted the assertion fifty times a day. The mere look of anxiety in his eyes gave him away, not to speak of the quickened pulse on the arrival of the doctor. He did his best to conceal his nervousness, but the maxim, "Give thy thoughts no tongue," was one that he found most difficult to follow, and remarks about the state of his health formed the chief feature of conversation. "I don't think I ought to feel like this, nurse. I never did before. Do you think it is anything unusual?" This was a question to which I became most accustomed, and which usually preceded a lengthy discussion of each new symptom, as to whether it was favourable or the reverse. On one occasion he had a slight pain in his side. "Nurse," he said, beckoning me to him with a trembling hand, "do you think there is any danger of pleurisy or inflammation? Does not this pain show that there is something the matter with my lungs?" I hastened to reassure him, but after this incident I was not surprised when, assailed by an irritable cough, more or less nervous, he inquired, with an agony of suspense in his eyes, if I did not think he had reached the earlier stages of phthisis.

Mr. Standish continually wanted his temperature taken, and I had to resort to all kinds of excuses in refusing to comply with his request, for if it had risen he delared that he knew he was much more feverish, if it had fallen he was sure that he was sinking fast. Sometimes I pretended not to hear the fatal question and rapidly began to speak of something else; at others I insisted that he must wait until I was less occupied, hoping by that time his thoughts might be turned into another channel.

He regarded the visit of the doctor with alternate delight and terror. It was always a great relief to be told how groundless were his nervous fears, and the doctor's reassuring optimism was his best tonic. But, on the other hand, his dread of an unfavourable verdict almost approached a nightmare. Whenever the doctor took me aside for a few minutes' discussion as to the treatment of our patient, I always felt that he was regarding our conversation with suspicion and that he concluded that it had for its basis the seriousness of his condition.

"Are you quite sure you told me everything that he said?" was the usual question the moment the doctor had left the house. "You are sure you are not keeping anything back. I am not at all nervous, and would prefer to know his entire opinion."

The visits of his friends were not an unmixed blessing. The favourite greeting was, "How do you think I am looking?" and his peace of mind depended upon their answer.

Those who were best acquainted with his temperament always assured him that he was better, but others, more sympathetic than discreet, would commiserate him on his appearance. I grew to dread the arrival of these pitying but foolish folk, for the rest of the day the burden of my patient's song was ever this, "So-and-so thought me looking very ill, nurse. Do you think that I am any worse?"

Of course he frequently thought that he was about to die—once or twice during the night he was absolutely sure of it. In great terror and distress he called me to his bedside, and told me that he was not certain as to the justice and wisdom of his will, and that he must alter it while there was yet time.

"You must send for the lawyer immediately," he declared. "Please let there be no delay. I feel certain that I cannot live many more hours."

I assured him that the lawyer should be sent for directly it was daybreak; and, his mind set at rest on that point, he drew a graphic and harrowing account of the future of his bereaved family, and gave me minute directions about the funeral and the place where he wished to be buried. By the time he had fixed on the exact spot for his grave he fell into a peaceful slumber, and when he awoke next morning he had lost the keen desire for the presence of his lawyer.

Another evening it was necessary to administer a sleeping draught, and then his anxiety knew no bounds. "Are you satisfied that you are not giving it to me too strong?" he inquired. "An over-dose might be fatal. One is always reading of such things. A friend of mine died a few years ago from that very cause, and he is only one of many." That idea having once occurred to his mind, he dwelt on it persistently, and began to relate all the cases of death through accidental poisoning of which he had ever heard.

He was always very careful about his diet, and the first occasion on which he partook of solid food was indeed momentous in his eyes. "Are you quite sure I may eat this fish?" he asked. "I did not hear the doctor mention it. Don't you think I had better wait until to-morrow, when you can ask him if he considers it wise? I should not like to take anything that might disagree with me or retard my recovery."

That was his constant cry; his burning desire was to get well, and in order to accomplish this end he submitted to any treatment, and was never rash or headstrong. In this respect he was an ideal patient; moreover, he was a very grateful one, and really believed that what was done was for his good. In spite of his gloomy forebodings Mr. Standish ultimately recovered—he will probably make many more changes in his will and his place of burial ere he dies.

### Appointments.

East Preston Union Workhouse.—Miss Margaret F. Rogers has been appointed Superintendent Nurse. She was trained at the London Homœopathic Hospital, Kensington Infirmary, L.O.S., and afterwards held appointments at Ticehurst Union, Sussex, and East Grinstead Union, Sussex.

Monsall Fever Hospital, Manchester.—Miss Annie L Cox has been appointed Lady Superintendent of Nurses. She was trained at Derbyshire Infirmary, has been attached to the Kent Nursing Institution, and for the last eight years has been lady superintendent of the Hull Royal Infirmary.

Down District Asylum, Downpatrick.—Miss Mary Harkin has been appointed Head Nurse. She was trained at Sir Patrick Dunn's Hospital, Dublin, and afterwards became sister in the same institution.

Kettering Union Workhouse Infirmary.—Miss Laura Errington has been appointed Charge Nurse. She was trained at Brownlow Hill Workhouse Infirmary, Liverpool, where she was probationer and charge nurse.

### MAKING ENGLISH BUTTER AT A KASHMIR HOSPITAL.

It is a fact worth recording that one of the nurses at the Hospital of Srinager, in Kashmir, makes excellent English butter. We are not surprised to learn that the many visitors to the place gladly avail themselves of the opportunity of obtaining some of the butter. The hospital itself is built at the foot of a hill, and its aspect being north it is comparatively free from the glare of the sun. The views rom the building are very fine, and combine a panorama of ake as well as a giant mountain.

### Everybody's Opinion.

[Correspondence on all subjects is invited, but we cannot in any way be responsible for the opinions expressed by our correspondents. No communication can be entertained if the name and address of the correspondent is not given, as a guarantee of good faith but not necessarily for publication, or unless one side of the paper only is written on.]

### A NURSE AT EARL'S COURT EXHIBITION.

In reply to "Curiosity" of August 19th, "Florence" writes: That the nurse at one of the stalls at Earl's Court Exhibition is there to explain to anyone interested the properties of the preparation sold. It is a new Queensland remedy for headache, toothache, &c., or for use in a vapouriser in respiratory trouble. It has a very pleasant odour, similar to that of Oak Leaf geranium.

### NURSES' READING SOCIETY.

Miss J. G. Moberly writes: "I am glad to say that I have been able to arrange with Mr. Glaisher (bookseller), of Wigmore Street, W., to start a lending library for nurses. Nurses will be able to join for next quarter. Mr. Glaisher most kindly gives us the lowest possible terms, and proposes making the subscription for each nurse 8s. a-year. For this each nurse will be able to borrow a book a quarter. In order to arrange such moderate terms, Mr. Glaisher has asked me to get two books quarterly, and to arrange that one half of the nurses should read one book, the other half reading the other book. The following quarter the books will be interchanged. I shall, of course, let each individual nurse know what book she is expected to read each quarter, and as at first only a yearly prize can be given, this division of books will not in any way affect the examination papers and marks. May I once more emphasize the rule that nurses writing to me for information about the society must enclose a stamped envelope if they wish me to reply to them. I have received more than one post-card requesting me to send the society's rules, and I have not replied to these cards because no stamp has been sent me."

### DO NURSES SMOKE?

"A Nurse Who Has Not Taken to it Yet" writes, in reply to "M. G. S.," August 19th: It is not because I think it wrong for nurses to smoke that I am expressing my thoughts upon the subject, and whether I consider it "mannish" or not is scarcely the point either; but I am inclined to think that it is more womanish than womanly to indulge in the habit. Do we nurses boast that our training does much to fortify our character, and then must we, of all people, fly to my Lady Nicotine to soothe our nerves, to prop up our good humour, to induce serenity of manner, or to smooth out our little crochets, as if we were mere men? We talk about our pleasures not so being numerous that we should be debarred from smoking. As a nurse, I find that my pleasures are quite as numerous as those of other people. Certainly, one has not always time for them, but they will generally keep, and thus become all the sweeter, like good apples. I am always sorry when nurses talk about themselves as if they lived in a perpetual state of self-sacrifice, and ought therefore to be allowed this or that indulgence to make up. I believe it is this ignoble sentiment of self-pity which has made many a nurse fall by little and little into habits of intemperance. If our training has done anything appreciable in the fortifying of our characters then we ought to be in a better position than other women to "bear the infirmities of the weak, and not to please ourselves."

### BREAKFAST IN THE BEDROOM.

"Alice" writes: My experience is that it is not usual to give nurses breakfast in the bedroom. I have never been asked to take my meals in the kitchen. I am a nurse of twelve years' standing, and during my nine years' experience in private nursing, with one or two exceptions I have been treated as a member of the family, and cannot say enough for the kindness and consideration I have received from all classes, rich and poor alike. As a rule I have been given a room to dress and undress in, and have not been expected to do either in my patient's. I agree with "Edith" that no one should enter the field of private nursing who does not love her work. The hours are longer than hospital work, and more tact and patience are required. So far my experience has been a very happy one, quite different to "Edith's." When people have been in trouble they have looked for help and sympathy, but I have also found most persons willing to put themselves to any trouble to give me pleasure. Nursing is from beginning to end a life of self-denial, and no woman should offer herself for training as a nurse unless she is prepared to do real hard work, and to practice incessant self-denial and patience. She must sympathise with the sufferer, put herself in the patient's place, be careful of his feeling, temper, &c., and think of self not at all. I wish that all nurses could look back on their nursing life with as much pleasure as I do on mine.

"Surprised" writes: I wish to make a few remarks on the paragraph by "Edith" on "Breakfast in the Bedroom." "Edith" says, "If a nurse refuses to take her meals in the kitchen they are served to her in her own, or her patient's room." My experience as a private nurse has been very different. I have never yet been requested to take my meals in the kitchen or in my patient's room. In my humble opinion it depends—now that there are so many ladies in our profession—upon each individual nurse whether she is treated "as one of the family" or not. And as for having "frequently had nowhere to bath or dress, eat, or sleep," except in the patient's room—all I can say is that "Edith's" lot must have been cast with particularly unrefined and selfish people. "Edith" goes on to assert—after explaining that she now works independently of any institution—"I make my own terms, and go nowhere where I am not received as one of the family and kindly treated." How can she possibly make these arrangements for her comfort beforehand? Does she interview the lady of the house, and stipulate that she only undertakes to nurse the patient under certain conditions, one being that she is "treated as one of the family"? Again, "I ask and obtain a high salary." Quite right, if it can be afforded. But after pointing out that she goes "nowhere" where she cannot command all these considerations, she ends her paragraph by saying, "It is a self-denying life, a giving up of all your own wishes (?) always doing as others please," &c.

### PRIVATE NURSING.

"A Member of the Guild of St. Barnabas" writes: Having read with much interest the correspondence in your columns on this subject, I feel I must say a few words in regard to both sides of the question. I myself am engaged at the present in private work, and I do not think some of your correspondents take the matter quite in the right light. In regard to the patients and their friends the position of the nurse is often a very difficult one. Sickness comes so suddenly upon us, that often a nurse arrives to find the house upset and the patient's friends all in a state of fluster and excitement. If she is quiet, sensible, and tactful, she will soon be able to restore order, and by her wise control of the sick room make them look up to her as a help, and not as a hindrance. So often nurses come into a house, and by their demands for themselves and their patients cause needless trouble and annoyance. I have frequently been told by those whom I have nursed of the trouble members of our profession have caused by their thoughtlessness, and in my own home we have had nurses who gave the servants much unnecessary work. Surely in the light of our Lord's teaching we should be proud to help bear the burdens of others, and to let our little light shine wherever we go. Nursing in private work is often hard and irksome; want of consideration may be shown, but with forethought much unpleasantness may often be avoided. As "An Old Nurse" says, we must take the rough with the smooth, and by putting ourselves in the background gradually become a help to those to whom we are sent. I am working for myself, but I do not leave off uniform, as I find (if simple and neat) it entitles one to respect, and it has never prevented my being on terms of equality with my patients or their friends. A life spent in helping to restore life and strength to others is surely too sound a work for such petty details as our wishes in regard to meals, dress, smoking, &c. Let us all rather try and remember that "Inasmuch as ye did it unto the least of these My brethren, ye did it unto Me."

### For Reading to the Sick.

Pray for one another! . . . The effectual fervent prayer of a Righteous man availeth much.—*S. James v.* 16.

Say not all useful work thou art denied!
Behold! Christ's censer waiteth at thy side.
He in compassion lets it down to thee,
Heap on thine incense! heap it full and free.
Pray for thy friends! that every deed of love
May be received and registered above.

Pray for the sick who suffer in all lands,
God's prisoners, laid in bonds by His own hands.
Pray for Crowned Heads, with all their weight of care,
For broken hearts, and all the sorrows there;
For the whole race which He has made His own,
For which He intercedes before His Throne.
—*C. M. Noel.*

The lonely sufferer is still a fellow-worker with Him. . . . A sleepless voice of Intercession unheard by man, but borne to God by a "surrendered soul," may bring strength to combatants wearied with a doubtful conflict.—*Wescott.*

Habitual prayer constantly confers decision on the wavering, energy on the listless, calmness on the excitable, and disinterestedness on the selfish. It braces the moral nature by transporting it into a clear, invigorating, unearthly atmosphere; it builds up the moral life insensibly, but surely; remedying its deficiencies and strengthening its weak points, till there emerges a comparatively symmetrical and consistent whole, the excellence of which all must admit, although its secret is known only to those who know it by experience. . . . Prayer makes men, as members of society, different in their whole bearing from those who do not pray. It gilds social intercourse and conduct with a tenderness, an unobtrusiveness, a sincerity, a frankness, and evenness of temper, a cheerfulness, a collectedness, a constant consideration for others, united to a simple loyalty to truth and duty, which leavens and strengthens society.—*Liddon.*

Why for the dead, who are at rest?
Pray for the living! in whose breast
The struggle between right and wrong
Is raging terrible and strong,
As when good Angels war with Devils.
—*Longfellow.*

Prayer diffuses a spirit of peace over our life. In prayer the soul gains repose. Then are the storms and passions of the heart silenced, the disturbance of its cares, anxieties, sufferings, and even of its joys cease. Fresh vigour and cheerfulness break forth upon us. As the bracing air of the mountains fills us with a sense of renewed power, so do we in prayer breathe an atmosphere of divine encouragement, and come forth from the inner sanctuary of communion with God to enter with new alacrity into external life.—*Liddon.*

My Redeemer and My Lord!
I beseech Thee, I entreat Thee,
Guide me in each act and word,
That hereafter I may meet Thee,
Watching, waiting,
Hoping, yearning,
With my lamp well trimmed and burning!

Interceding,
With these bleeding
Wounds upon Thy Hands and Side,
For all who have lived and erred,
Thou hast suffered, Thou hast died,
Scourged and mocked and crucified,
And in the grave Thou hast been buried!

If my feeble prayer can reach Thee,
O, My Saviour! I beseech Thee,
E'en as Thou hast died for me,
More sincerely
Let me follow where Thou leadest!
Let me, bleeding as Thou bleedest,
Die, if dying I may give
Life to one who asks to live,
And more nearly,
Dying thus, resemble Thee!
—*Longfellow.*

### Notes and Queries.

**The contents of the Editor's Letter-box have now reached such unwieldy proportions that it has become necessary to establish a hard and fast rule regarding Answers to Correspondents. In future, all questions requiring replies will continue to be answered in this column without any fee. If an answer is required by letter, a fee of half-a-crown must be enclosed with the note containing the enquiry. We are always pleased to help our numerous correspondents to the fullest extent, and we can trust them to sympathise in the overwhelming amount of writing which makes the new rules a necessity.**

**Every communication must be accompanied by the writer's name and address, otherwise it will receive no attention.**

*Training Schools.*

(189) Will you kindly tell me the advantage of paying a premium to enter a hospital as probationer? (2) and also which of the London hospitals is considered the best training school?—*Violet.*

There are really no practical advantages now in paying a premium to be taught nursing. The training is identical for both paying and non-paying probationers, and in very few instances do the paying probationers enjoy any extra privileges or comforts. (2) It is quite impossible to say which is the best training school amongst so many excellent institutions. Judging by the number of applications received by the matrons of all the well-known hospitals, you will be lucky to be accepted as probationer by any one of them.

*Deafness.*

(190) Three or four years ago I saw a "Black Fan" which was used by a lady who was very deaf. I have never seen one since, nor have I heard nor been able to find out anything about them. The father of one of my friends is painfully deaf, and though he has an ear trumpet and tried various other things, none of them seem to me to answer as well as the "Black Fan," which was put just between the teeth. I should be so much obliged if you could help me to find out about it; where we could get one; and the price.—*M. R.*

The black fan alluded to is probably one sold by Hawkesley, of Oxford Street, which is very useful in some cases. But the best plan would be to consult a good aural surgeon, as there are many things to be considered in the treatment of deafness.

*Imbeciles.*

(191) I should be glad to know if there is an institute where a poor, helpless, imbecile child could be received without payment. A doctor in London advised its being sent to Darenth Asylum, but on making inquiries I find it is only for those living within a specified distance, which renders it out of the question.

Apply the Secretary of the Earlswood Asylum for Idiots and Imbeciles, Redhill (office, 36, King William Street, London, E.C.) The terms of admission are by annual payments or by election of subscriber.

*Male Nurses.*

(192) Could you tell me if there are male nurses trained for private work (medical and surgical), to what institution do they belong, and what is their charge per week?—*Nurse Question.*

Male nurses are trained at the National Hospital for the Paralysed and Epileptic, Queen's Square, Bloomsbury, W.C. Other male nurses have frequently received their training in lunatic asylums. They earn from £2 2s. to £3 3s. a week. You will find reliable agencies for supplying male nurses in our advertisement columns.

*Open Air Treatment.*

(193) Will you kindly tell me of a concise pamphlet on the open-air treatment of consumption, and the Nordrach feeding system?—*B. E. M.*

"Open-Air Treatment of Phthisis," by R. Greene, price 6d., published by the Scientific Press (Limited). Recent numbers of the *Practitioner* (Cassell and Co.) contain a good deal of useful information on the subject.

*Comparisons.*

(194) Will you kindly give me the names of the principal big London hospitals, and say in what rank they stand? Does the "London" stand first?—*Florence.*

Florence sets us an invidious and ungrateful task, which, thanks to the many and varied excellences of the London hospitals, it is fortunately impossible to undertake. If Florence had heard students or nurses representing all hospitals discussing the merits of their own special institution she would speedily have come to the conclusion that each hospital was the best in the world, and that none of the others could hold a candle to it.

*Stewardess.*

(195) Would you kindly tell me the best way to obtain a post of stewardess on board the P. and O. Company or any other of the principal mail lines? I have trained in general nursing and maternity work, and am a member of the Royal Pension Fund for Nurses.—*M. A. C.*

It is difficult to obtain such appointments without the interest of some of the companies' officials. You can only apply to the secretaries of the various shipping companies, enclosing a short list of your qualifications for the work and the names of one or two good referees.

*Gynæcological Nursing.*

M. D. B. writes in reply to F. C. P.: "Notes on Gynæcological Nursing" by Dr. Hellier is a very good little book, and it is published at, I think, 1s. 6d., by J. and A. Churchill.

### ANSWERS REQUESTED.

*The Greek Hospital, Alexandria.*

A nurse would be glad of any particulars relating to this hospital.

### Travel Notes.

By the Travel Editor.

### XXXIV.—BRUGES.

I AM directing my attention now particularly to the description of places that shall be inexpensive and suitable as holiday resorts for nurses. They as a class have shown the most appreciation of this section of the "Nursing Mirror," and I shall always be glad to help them in any way possible. I am also open to any suggestions. Should anyone like a detailed description of any locality likely to be popular with nurses, I shall be happy sometimes to give an article on the place if suitable; at any rate do not hesitate to write to me if I can be useful to you in any way. I have been a great traveller myself, and it gives me great pleasure to help others to the same enjoyment. The place we are considering to-day is not a good centre for cyclists because the roads of North Belgium are pitched in a manner that makes wheeling very fatiguing. Its chief attractions are, for those who like old-world cities, mediæval architecture, art treasures, and a thousand historical recollections of the past. The Flemish cities abound in interest for the artist and the photographer, but the surrounding country is flat and somewhat uninteresting, and for those who like to take long walks and enjoy fine scenery, I consider North Belgium entirely unsuited.

### Expenses of the Journey.

Second class return via Ostend is £2 0s. 1d. Bruges is close to Ostend, so the journey is a particularly easy and non-fatiguing one, and being so short, attendant expenses are small. There is still a cheaper way, if you are not pressed for time, by the Steam Navigation Company's boats from Tower Bridge to Ostend, first class return, 10s. 6d., second ditto, 9s., and very comfortable they are. I have twice gone by them.

### Accommodation in Bruges.

It is not at all an expensive place, and the hotel accommodation is very good. The Hotel du Sablon will take you for seven francs per day on the third floor, and the Panier d'Or for still less, I believe, by arrangement. It is always better to write beforehand; you are invariably treated with more consideration. It is still a quiet place, in spite of its popularity with the English, and if you go in the early summer, or indeed, any time but during August and September, everything is cheap, especially in the markets.

### The Sights of Bruges.

First among these comes the grand old belfry with its silver chimes or carillon as they are called. I am one of those unfortunately constituted people who on hearing English bells yearn to throw my head back and howl like a dog to express the mysterious misery that afflicts me, but the effect of Belgian carillons is totally different. They give me the most unfeigned pleasure; whatever the strange influence, which no one can explain, but which so many feel to their cost with ordinary bells, is quite absent. This harmonious chime goes every quarter of an hour, and has a most soothing effect. The mechanism resembles that of a large musical box, and the ringer plays on a species of keyboard with leathern thimbles on his fingers. If you chance to be ascending the hundreds of steps in the interior when the carillon starts the effect is rather terrific, the tower seems to rock around you and to be about to come crushing down and annihilate you in the ruins. The tower stands over the centre of the meat market, and is a most graceful sexagonal erection; the best view is that seen coming up the Rue St. Amand, a delightful old street but little modernised; at the corner is an old house considered to be that in which our Charles II. lived when an exile.

### The Hospital of St. Jean.

This is chiefly interesting as being the home of Hans Memling's finest work. As one looks at the wonderful miniature-like figures that glow like jewels and are as fresh as when they sprang to life under the master's hand more than four hundred years ago, one thinks of the wounded and weary soldier who in 1480 came a suffering suppliant to the convent gates, and being tenderly cared for by the nuns, rewarded them by a present of some of his finest paintings. The most interesting work of the master is the "Chasse de Ste. Ursule," which represents the history of the saint and her 11,000 virgins.

### The Hotel de Ville, Palais de Franc, and Chappelle du Saint Sang.

These buildings stand in a picturesque mass to the east of the market place. There is a vaulted passage under the Hotel de Ville which leads through to the canal Vert. From here, looking back on the town, is one of the best known views in Bruges. Close at hand is the Palais de France, and in the distance the belfry and cathedral.

### The Cathedral and Notre Dame.

I feel but languid interest in the cathedral, though it is a fine building, but Notre Dame holds my affections. The exquisite tombs of Charles the Bold and of his daughter, which are in a closed chapel to the right, are worth going all the way to Bruges to see alone; the wonderful workmanship, the rich, dim colouring, and the grace of the recumbent figures are a perpetual delight. Remember that the churches are always closed from noon till two. There is a third church in Bruges worthy of notice from its being supposed to contain an imitation of the Holy Sepulchre; it was founded by a burgomaster, who twice travelled to the Holy Land to make sure of his facts. Not far from here is the old bowling alley and archery ground of St. Sebastien, where our Charles II. beguiled his many idle hours. Do not omit a visit to the Béquinage, where uncloistered nuns live, and manufacture lace, tapestry, &c. Near the Béquinage is the Minnewater, one of the prettiest spots in the town; the bridge is long, and has a tower supposed to be haunted by the spirit of a love-sick maiden who drowned herself in the sheet of water below.

---

### TRAVEL NOTES AND QUERIES.

GHENT (Amateur).—It is not quite so suitable a place as Bruges for residence, and it is not calculated for cyclists; the roads of North Belgium are many of them cobbled, and the scenery is in no way beautiful. South Belgium, on the contrary, presents great attractions to cyclists. See answer to "Etoile." Belgium is but a small country, and you can comfortably go from point to point on your machine, but long excursions for the sake of beauty of scene are not to be had.

SCOTLAND (Colonial).—No; I fear you cannot do it on the sum you have at your disposal. It is a very expensive place, and even at small temperance hotels you cannot reckon on less than nine shillings a day. In very outlying regions, where you might naturally expect lower terms, the reverse is the fact, owing to the want of competition, and the difficulty of getting provisions. You will be fortunate if you can limit your expenses one day with another to ten shillings per diem.

THE ARDENNES (Etoile).—Splendid cycling country. Dinant is the best centre. From there you can see the whole Walloon country. The chateaux of Walzin, Veve Celles, &c., are near to Dinant, and the beautiful country round La Roche and Rochefort is easily explored. An article on this locality appeared in the "Mirror" in the spring. Terms in the Ardennes, even in the season, may be kept to seven or eight francs, or less by arrangement.

PAU (Erin).—The end of October is a good time to start, and you can comfortably remain at Pau until April. It is an expensive place. If you settle to go let me hear again, and I will give you some addresses. If you elect to spend the summer in the Pyrenees you have a great choice of mountain resorts—Eaux Chandes, Eaux Bonnes, Luchon, Argeles, &c. Two winters spent in Pau and the intervening months in the mountains usually is found extremely beneficial.

THE CAPE (Querist).—Your letter was addressed entirely wrong, hence there has been considerable delay. The weather to be expected will be warm on the whole; take light woollen clothes, which are always safest, and plenty of warm wraps for bad days. Weather at the Cape equable and rather warm. Keep to light woollen underclothing, and have some thin dresses of wool and also of cotton or washing silk. I do not think there will be any possible objection to uniform. As to your fee I should charge exactly the same as at home, unless expense is an object to the patient, when you might charge a little less. The question of fees is not exactly in my department; if you would like another opinion write to the Editor separately on the subject, but be careful of the address.

